# Internalization of athletic body ideal as a mediating variable between family influence and body image of young women. A cross-cultural study of polish, Italian, and Ukrainian women

**DOI:** 10.3389/fpsyt.2023.1136079

**Published:** 2023-03-23

**Authors:** Bernadetta Izydorczyk, Kaja Głomb, Barbara Bętkowska-Korpała, Tetiana Yablonska, Nataliya Bulatevych, Renata Opałka, Sebastian Lizińczyk, Katarzyna Sitnik-Warchulska, Bartosz M. Radtke, Urszula Sajewicz-Radtke, Małgorzata Lipowska

**Affiliations:** ^1^Institute of Psychology, Jagiellonian University, Krakow, Poland; ^2^Institute of Applied Psychology, Jagiellonian University, Krakow, Poland; ^3^Collegium Medicum, Jagiellonian University, Krakow, Poland; ^4^Faculty of Psychology, Taras Shevchenko National University of Kyiv, Kyiv, Ukraine; ^5^Central Board of Prison Service, Ministry of Justice, Warsaw, Poland; ^6^Laboratory of Psychological and Educational Tests, Gdańsk, Poland; ^7^Institute of Psychology, University of Gdansk, Gdansk, Poland

**Keywords:** musculature in women, body image, westernization, family role, sports body standards

## Abstract

**Introduction:**

Our aim was to analyze the strength of the family’s influence on the internalization of the ideal of an athletic figure and, consequently, on the multifactorial image of the body, from the perspective of intercultural differences

**Methods:**

A total of 488 healthy women aged 19–26; of Polish (154), Ukrainian (228), and Italian (106) took part in the study. The Sociocultural Attitudes Towards Appearance Questionnaire (SATAQ-4) and the Multidimensional Body-Self Relations Questionnaire (MBSRQ 69) were used to measure athletic ideal internalization and family pressure. The body image of Ukrainian, Polish, and Italian women depends both on the degree of internalization the ideal of an athletic figure and influence of the family

**Results and Discussion:**

The research also suggests significant differences between the three populations, which may suggest cultural differences between young women living in Eastern, Central, and South European countries.

## Introduction

The search for scientific evidence confirming the existence of sociocultural and family predictors explaining the specificity of the pursuit of the perfect body appearance and its musculature is a subject that increasingly inspires contemporary psychological research in nonclinical and clinical populations (1). The athletic or muscular body ideal has recently been debated as a new and increasingly common standard of female beauty and is a potential alternative to the slim figure ideal, the internalization of which is often associated with body dissatisfaction and risky eating behavior [e.g., (2–7)]. However, the lack of consistency in the research on this issue to date prevents a precise, scientific recognition of how the sociocultural standards of the ideal of an athletic or muscular figure promoted in culture may affect the way one perceives and experiences one’s own body. Another important question in this context is the role of the family in internalizing this new ideal of beauty and its impact on the overall body image—understood as a multifactorial construct describing the self-esteem of body appearance, physical fitness, health, as well as the individual’s focus on body weight and the fear of gaining weight. This is important because the family, as a basic social group and one of the most important sources of value, is traditionally considered one of the risk factors for eating pathology (e.g., social group) (8, 9). Of course, other factors also play a role in the process of shaping the body image in adolescents and young adults, e.g., peers or partners of a romantic relationship (10); however, the authors of this article focused on the search for the role of family factors—on the body musculature of young women, which is much less studied.

### Athletic body standards in women

The image of an ideal female body, strongly promoted in the media in Europe (11) and the United States (12), complies with the standards of excessive striving for body thinness and, more and more often, with the standards promoting the requirement to have an athletic body shape. The trend of promoting athletic body standards observed in contemporary culture is also reflected in the dynamic development of the fitness industry and gyms, i.e., places where physical activity is promoted. The dissemination of standards that strengthen a person’s desire to have an athletic body shape may, on the one hand, strengthen pro-health behavior toward the body, i.e., promote health and physical fitness of the body, but on the other hand, may favor the development of anti-health behavior toward the body (13).

Research on the subject of the standards of an ideal fit and muscular body was more often conducted on male populations in various European countries (14–17) or the American male population (18, 19). There are also cross-cultural studies covering the populations of men from European, American, and African countries (20) or men of American and Latin origin (19). Research on body image was also carried out in the population of athletes, i.e., those most interested in pursuing the ideals of the athletic body (21).

Fewer studies have been conducted on women, who are traditionally tested for the pursuit of thinness rather than athleticism. Although there are few studies, their results seem to be consistent. On the one hand, internalizing the ideal of an athletic body can be as harmful as internalizing the ideal of a lean body and may be associated with effects such as eating disorders (22, 23), excessive exercise (22, 24, 25), objectification (11), and body dissatisfaction (24, 26). On the other hand, some studies suggest that the internalization of the sporting ideal may not be related to body dissatisfaction (25, 27). Moreover, when internalized in isolation (without the ideal of a lean body), the athletic ideal may be considered a protective factor (28). The presence of photos of athletic women in the media may also be beneficial for young women’s self-perception (29).

The literature review suggests the inconsistency of models describing the influence of sociocultural factors on the internalization of the athletic figure ideal. While peer pressure and family and media pressure are often seen as key variables in internalizing the slim ideal, causing body dissatisfaction and, consequently, eating disorders (Tripartite Influence Model; see (30), research into internalizing the athlete ideal hardly confirms this hypothesis. For example, Ramme, Donovan, and Bell (27) verified the adequacy of the Tripartite Influence Model in relation to the ideal of an athletic body and found no relationship between sociocultural influences and the internalization of the ideal of an athletic body. In turn, Donovan, Uhlmann, and Loxton (24) showed a relationship between sociocultural pressure and the internalization of the athletic ideal, but the coefficients in their model were very small; hence, the significance of the path could be the result of a large sample size.

It is true that there are few cross-cultural studies in the literature on the measurement of attitudes toward muscle and tissue in women. Examples include the studies by Gray and Frederick (31) on the population of women and men of the American and Caribbean population and research by Ramme, Donovan, and Bell (27). However, it is difficult to identify cross-cultural studies related to the European population in which the study verified the influence of the family on body image (defined not only as the attitude toward the body and its appearance but as a multi-factor emotional and cognitive structure describing the multi-faceted experience of the body and behavior taken pursuing it).

### Intercultural specificity of the family role in creating the appearance standard

Due to the insufficient number of intercultural studies relating to and comparing the European population and defining body image as a multifactorial construct, as well as considering the specificity of the upbringing of young women from this cultural area, the authors decided to refer, in their own research, to the most important questions about the relationship between the internalization of the ideal of an athletic figure, family and body image.

An analysis of culture-specific factors influencing body image can enrich the discussion of nonspecific risk factors for eating pathology. As Moleiro (32) emphasizes, “In a globalized world, it is currently widely recognized that it is the cultural context that defines (mal) adjustment of human behavior, which includes how people usually behave, think, feel, and relate in social interactions.” The authors believe that for disorders with sociocultural risk factors—and the pathology of eating is one of them—research should consider intercultural differences in order to integrate a cultural perspective into appropriate psychological interventions as well as in planning appropriate prevention.

### The current study: Poland, Italy and Ukraine as countries with different roles of the family in the process of internalizing beauty patterns

To empirically verify the relationship between the interesting variables, research was carried out on 3 nonclinical samples—healthy women (with the same age and BMI index)—from Poland, Ukraine, and Italy. The intention of the sample selection was, among others, to examine the differences but also similarities in terms of the specificity of family influences on body image in young women brought up in families living in the Eastern (Ukraine), Central (Poland), and Western (Italy) European countries. On the one hand, striving to achieve the ideal of an athletic body and its internalization may be an expression of the implementation of pro-health behaviors of physical activity, but also, when applied excessively and restrictively, it may lead to the consolidation of bigorectic tendencies (behaviors).

The study undertaken by the authors of this article aims to analyze the strength of the family’s influence on the internalization of the ideal of an athletic figure and, consequently, on the multifactorial image of the body. The study of the family role seems to be particularly important in the analysis of the cultural specificity of the studied populations. The selection of women brought up in families and living in Ukraine, Poland and Italy reflects the potential degree of intensity of the influence of Western culture: Italy, the Western European country that has undergone “westernization” for the longest time (33); Ukraine, an example of Eastern Europe with a relatively short history of political and cultural independence; and Poland, located between them.

Even though these countries are at different stages of political and social transformation, all three seem to be influenced by Western culture today. However, what is particularly important, in all three countries, is that the family plays a large role in shaping the identity of young people, their system of values, and the transmission of attitudes and beliefs [for Italy, see, e.g., (34, 35), for Ukraine, see, e.g., (36)]. Therefore, it seems possible that the role of the family in the context of body image research may be similar in these three populations. At the same time, due to the socio-political differences between these nations, the degree of internalization of the new, mainly Western standard of beauty may vary. It seems possible that, especially in the case of Ukraine, but also Poland, i.e., countries with a less ethnically diverse society, the ideals of the female figure may be more traditional and standardized.

## Methods and materials

### Operationalization of variables

**Figure 1:**
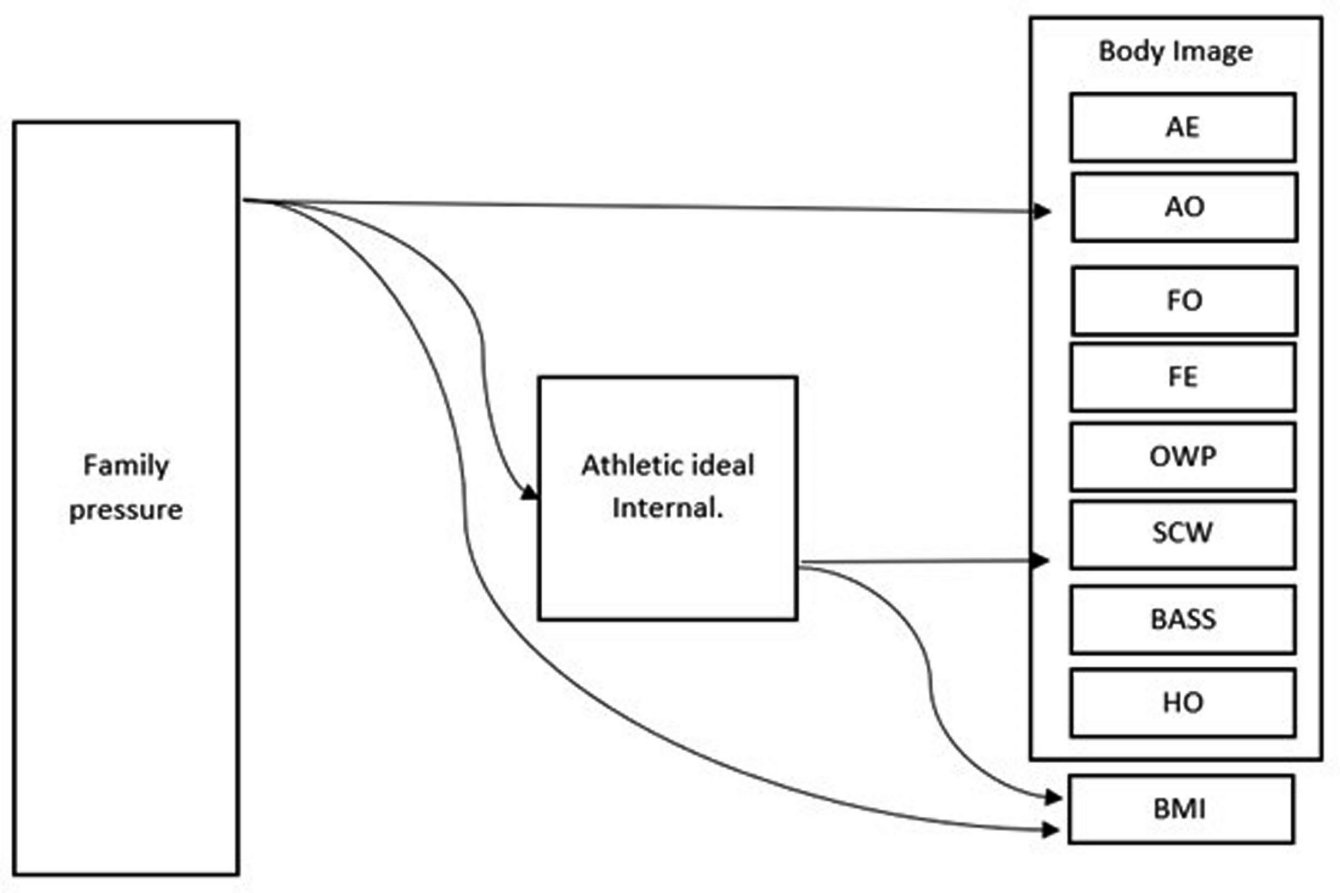
Research model: family pressure and body image in young women. The role of internalizing body musculature standards as a mediator between family pressure and the experience of body image. AE, appearance evaluation; AO, appearance orientation; FO, fitness orientation; FE, fitness evaluation; OWP, overweight preoccupation; SCW - self classified weight; BASS, body area satisfaction; HO, health orientation; BMI, body mass index.

The authors of this article, referring to the Tripartite Influence Model (30) and research on the analyzed issue, investigated to what extent the need to strive for the ideal body musculature facilitates the influence of family pressure on the body image of young women brought up in European culture, as well as samples of Polish, Ukrainian, and Italian women (Figure 1).

According to the research model, the explanatory variable was defined as family pressure. The variable explained was defined as body image and the intermediary variable as athletic ideal internalization.

Contrary to studies that treat body image as a uniform and one-dimensional variable, the authors define body image as an emotional and cognitive attitude toward one’s own physicality that can be expressed in four aspects of self-esteem: (1) body appearance; (2) assessment of physical fitness and focusing on the athleticism of the body; (3) body health evaluation and orientation; and (4) concentration on body weight and fear of gaining weight. This understanding of body image allows for a more detailed examination of the relationships between the studied variables and potentially allows for the formulation of predictions about the development and progression of disorders.

Family Pressure is a variable that describes the influence of the family environment communicating standards about an ideal, beautiful body. The influence is associated with direct comments on the appearance and by modeling attitudes, fears, and behaviors regarding the body. The study also monitored body mass index (BMI). To measure BMI, participants completed a questionnaire in which they were asked, *inter alia*, for height and weight. BMI was obtained by dividing the body weight in kilograms by the square of the height in meters.

Athletic Ideal Internalization is a variable describing the internalized pursuit of the standard of beauty of the female body, in which the main goal is to achieve an athletic figure with clearly defined muscles.

As a result, the following research questions were formulated:

1. Are there differences in the strength of family pressure, the pursuit of the ideal body musculature and individual aspects of body image (self-esteem, physical fitness, body health and focus on body weight) between the populations of young Polish, Ukrainian, and Italian women?
2. Does the internalization of the ideal of an athletic body shape mediate the relationship between family messages and body image in Polish, Ukrainian, and Italian women? If so, how?

#### Study participants

The survey was conducted online from January 2020 to January 2021 among young women from three European countries: Ukraine, Poland, and Italy, as a part of an international research project registered in Protocol Registration and Results System—ClinicalTrials.gov *blinded for review*. In total, it was planned to survey 600 young women—200 women from each country. Due to limitations caused by the COVID-19 pandemic, incorrectly completed psychological tests, and the presence of factors inconsistent with the inclusion criteria for the sample, 20 Polish and 20 Italian women were excluded from the study. No Ukrainian women were excluded from the study. As a result, 3 groups of women aged 19–26, with a BMI within the normal range for their age of life, were included in the statistical analyses, including 228 Ukrainian. The most important descriptive statistics of the demographic data of the studied groups are presented in Table 1.

**Table 1:** Descriptive statistics of the most important sociodemographic data.

	Statistics *M* (SD)		
	Ukrainian	Polish	Italian
Age (years)	20.34 (2,90)	22.17 (2.60)	24.23 (1.85)
Height (cm)	172,97 (9,44)	166.21 (6.29)	165.46 (6.04)
Weight (kg)	65.79 (14.62)	62.46 (14.42)	58.54 (9.83)
BMI	21.85 (3,95)	22.58 (4.82)	21.38 (3.42)

The criteria for inclusion in the group of respondents were: healthy women aged 19–26; of Polish, Ukrainian, or Italian nationality; raised and living in Poland (Polish women group), Ukraine (Ukrainian women group), or Italy (Italian women group); and having normal BMI.

On the other hand, the exclusion criteria were: the presence of health problems reported in the survey in the area of mental and somatic health, including physical disability and serious bodily injuries, dysmorphophobia, psychoses, depression, personality disorders associated with various forms of self-harm, and suicide attempts.

All subjects were presented with the purpose of the study, their consent was asked, and they were informed that participation in the study was voluntary and anonymous. The protocol of this study was approved by the *blinded for review* (decision no. *blinded for review*.). The questionnaires that formed this study took around 10 min to complete. We collected several different kinds of information using the *Sociocultural Attitudes Towards Appearance Questionnaire,* the *Multidimensional Body-Self Relations Questionnaire* and questionnaire containing sociodemographic data.

### Research methods

#### The sociocultural attitudes toward appearance questionnaire

The sociocultural attitudes towards appearance questionnaire (SATAQ-4) was used to measure athletic ideal internalization and family pressure. The SATAQ-4 is the latest version of the sociocultural attitudes questionnaire, which takes into account the Tripartite Influence Model (Keery et al., 2004), which assumes the existence of three basic sources of sociocultural pressure: peers, family, and the media. The method consists of 22 items that are rated on a scale from 1 (Definitely Disagree) to 5 (Definitely Agree). The items are grouped on five scales: Internalization—Thin/Low Body Fat, Internalization—Muscular/Athletic, Pressures—Family, Pressures—Peers, and Pressures—Media (Schaefer et al. 2015). Two scales were used in the research: Pressures—Family and Internalization—Muscular/Athletic. The Polish language adaptation of the questionnaire (not yet published) was prepared by a team led by *blinded for review*. Sworn translators of the English language translated the items into Polish and then independently back-translated them into the original language. Then, a pilot study was carried out on a group of 167 people (146 women). Factor analysis showed internal consistency of the questionnaire. This allowed the original division of scales to be preserved. Cronbach’s alpha test demonstrated the internal reliability of the test.

The Italian and Ukrainian versions were created by translating from the original language into Ukrainian and Italian and then translating it back and comparing the translated items. Ultimately, the Polish, Ukrainian, and Italian versions consisted of 22 items that respondents answered on a five-point scale from 1 (strongly disagree) to 5 (strongly agree). The values of Cronbach’s alpha reliability coefficients for individual subscales of the SATAQ questionnaire, calculated based on the authors’ own research, are presented in Table 2.

**Table 2:** Reliability (Cronbach’s alpha) analysis of the subscale and the entire SATAQ-4 questionnaire in the original version and in Polish adaptation in the group of Ukrainian, Polish, and Italian women.

	Number of items	Ukrainian (*n* = 228)	Polish (*n* = 154)	Italian (*n* = 106)
SATAQ-4	22	0.850	0.768	0.879
THIN	5	0.850	0.859	0.905
ATHLETIC	5	0.849	0.912	0.935
PF	4	0.849	0.923	0.862
PP	4	0.909	0.924	0.894
PM	4	0.948	0.914	0.916

THIN, Internalization–Thin/Low Body Fat; ATHLETIC, Internalization–Muscular/Athletic; PF, Pressures – Family; PP – Pressures – Peers; PM, Pressures – Media.

#### The multidimensional body-self relations questionnaire

The Multidimensional Body-Self Relations Questionnaire (MBSRQ 69; (37) was used to examine body image. It allows the subject to explore the various dimensions of experiencing their own body (self-esteem, physical fitness, health and concentration on body weight and concerns about gaining weight). The test subject refers to each of the 69 items on a scale from 1 to 5. The higher the score, the greater the satisfaction with body areas and their functions. The test includes the following scales: appearance evaluation (AE), appearance orientation (AO), fitness evaluation (FE), fitness orientation (FO), health evaluation (HE), health orientation (HO), illness orientation (IO), body area satisfaction (BASS), overweight preoccupation (OWP), and self-classified weight (SCW).

In Poland adaptation of MBSRQ by Brytek-Matera and Rogoza (38) was used. In the studies of the Ukrainian group, the linguistic adaptation of the tool was made. Sworn translators translated the English version of the MBRSQ into Ukrainian and then independently translated it into English (original for MBRSQ). The Italian version was used in the Italian group. In the research of the authors of this article, Cronbach’s alpha obtained in the Ukrainian, Polish, and Italian samples is presented in Table 3.

**Table 3:** Reliability analysis (Cronbach’s alpha) of the subscale and the entire MBRSQ in the studied groups of Ukrainian, Polish, and Italian women.

	Number of items	Ukrainian (*n* = 228)	Polish (*n* = 154)	Italian (*n* = 106)
MBSRQ	69	0.850	0.912	0.871
AE, Appearance evaluation	7	0.723	0.918	0.857
AO, Appearance orientation	12	. 789	0.804	0.801
FE, Fitness evaluation	3	0.775	0.839	0.654
FO, Fitness orientation	13	0.775	0.950	0.854
HE, Health evaluation	6	0.782	0.801	0.615
HO, Health orientation	8	0.783	0.722	0.565
IO, Illness orientation	5	0.565	0.592	0.656
BASS, Body area satisfaction	9	0.825	0.824	0.807
OWP, Overweight preoccupation	4	0.706	0.720	0.708
SCW, Self-classified weight	2	0.735	0.865	0.736

The remaining data were obtained using a questionnaire. It included the following information: gender, age, height, and weight; health and mental disorders such as physical disability and serious bodily injury, dysmorphophobia, psychosis, and depression; personality disorders associated with various forms of self-harm; and suicide attempts.

SPSS package software for windows 24 was used for statistical calculations. AMOS v.24 software was used to calculate the structural equations (SEM).

## Results

### Characteristics of the intensity of body image indicators and the internalization of athletic body posture standards (comparative analysis of polish, Ukrainian, and Italian women)

#### Differences between the three populations

To determine the differences in the levels of the studied variables in the three samples, one-way analysis of variance was used. A *post hoc* Bonferroni test was used to identify intergroup differences. The description of the mean values for individual variables and the measurement of the significance of the differences are presented in Table 4.

**Table 4:** One-way analysis of the variance of the studied variables for Ukrainian, Polish, and Italian women.

Variable	Ukrainian (1) M	Polish (2) M	Italian (3) M	ANOVA (p) Significant Bonferroni *post hoc* test results
BMI	22.20	22.58	21.38	*F* (689) = 1,0; *p* > 0.05
AE, Appearance evaluation	19.15	17.97	18.47	*F* (689) = 13.4; *p* < 0.001 (1) > (2); (1) > (3)
AO, Appearance orientation	16.26	14.77	15.62	*F* (689) = 15.6; *p* < 0.001 (1) > (2); (2) < (3)
FE, Fitness evaluation	18.54	19.66	17.94	*F* (689) = 15.5; *p* < 0.001 (1) < (2); (2) > (3)
FO, Fitness orientation	38.90	38.17	38.38	*F* (689) = 1.6; *p* > 0.05
HE, Health evaluation	31.06	28.67	29.47	*F* (689) = 27.8; *p* < 0.001 (1) > (2);(1) > (3)
HO, Health orientation	37.13	34.27	35.97	*F* (689) = 21.1; *p* < 0.001 (1) > (2);(2) < (3)
IO, Illness orientation	9.48	11.18	10.16	*F* (689) = 11.0; *p* < 0.001 (1) < (2)
BASS, Body area satisfaction	6.06	6.26	6.13	*F* (689) = 1.2; *p* > 0.05
OWP, Overweight preoccupation	33.35	28.68	29.09	*F* (689) = 45.7; *p* < 0.001 (1) > (2);(1) > (3)
Inter. Thin low body fat	12.43	16.96	14.25	*F* (689) = 51.16; *p* < 0.001 (1) < (2);(1) < (3);(2) > (3)
Athletic ideal internal	14.48	11.79	14.10	*F* (689) = 16.90; *p* < 0.001 (1) > (2);(2) < (3)
Family pressure	7.08	9.05	9.32	*F* (689) = 20.59; *p* < 0.001 (1) < (2);(1) < (3)
Peers pressures	6.13	7.49	8.60	*F* (689) = 20.11; *p* < 0.001 (1) < (2);(1) < (3)
Media pressures	8.96	12.51	12.17	*F* (689) = 34.77; *p* < 0.001 (1) < (2);(1) < (3)

The table shows the mean values of the studied variables and the results of ANOVA tests.

The analysis showed significant differences between the groups in appearance evaluation, fitness evaluation, health evaluation, fitness orientation, health orientation, overweight preoccupation, body area satisfaction, athletic ideal internalization, and family pressure. In the Polish sample, the highest levels of health evaluation, overweight preoccupation, and family pressure and the lowest level of athletic ideal internalization were recorded, while in the Ukrainian group, the highest levels of appearance evaluation, fitness evaluation, fitness orientation, health evaluation, and body area satisfaction were recorded. One-way ANOVA showed that BMI, appearance orientation, and self-classified weight did not differ significantly between the studied groups.

The analysis showed significant differences between the groups in appearance evaluation, fitness evaluation, health evaluation, fitness orientation, health orientation, overweight preoccupation, body area satisfaction, athletic ideal internalization, and Peers Pressures and Media Pressures.

### Characteristics of the influence of family messages and the standards of The ideal of an athletic body (musculature) on the body image of polish, Ukrainian, and Italian women (comparative analysis—modeling of structural equations)

*To answer the second research question* (whether, and if so, how, the internalization of the ideal of an athletic body shape mediates the relationship between family messages and the dimensions of body image in Polish, Ukrainian, and Italian women), a multidirectional analysis of structural equations was carried out.

It was checked whether family pressure has a direct impact on body image in the studied groups or whether this influence is mediated by sociocultural standards regarding athletic ideal internalization of the female body. To explain the relationships between the variables, an integrative model of hypothetical relationships between the variables was tested and verified by the analysis of structural equations (see: Figure 1).

The exploratory path model was based on modification indices and reduced (to improve readability) by irrelevant paths (*p* > 0.05). From the prediction of high goodness of fit indices of the model, it can be concluded that it well represents the correlation matrix underlying the empirical data. All indices such as Chi^2^, CFI, and RMSEA, prove that the model is well adjusted to empirical data. The Chi^2^ RMSEA indices were at a good level of fit, and the GFI index indicated a very good level of fit. Fit indices for each study group can be found under each figure.

**Figure 2:**
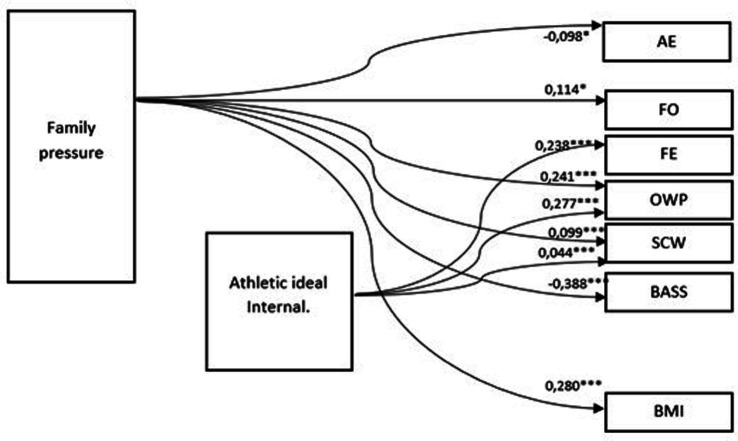
Path model (SEM) for young Ukrainian women. *N* = 228; Chi^2^ = 0.16; df = 1; *p* > 0.05; RMSEA = 0.010; CFI = 0.997. * = *p* < 0.05; ** = *p* < 0.01; *** = *p* < 0.001 (two tailed test).

### Characteristics of the influence of family messages on the internalization of the standards of athletic style (musculature) and on the general body image in the Ukraine group

Analyzing the relevant path models and path coefficients presented in Figure 2, it can be indicated that the internalization of the athletic ideal affects some components of body image, but this variable is not affected by family pressure. Thus, the internalization of the athletic ideal does not mediate between the social norms related to family messages and the standards of female beauty and for the women studied.

For the readability of the graph, only significant paths are presented, and graphical markings of the residuals are omitted.

As far as the internalization of the ideal of an athletic figure is concerned in the case of young Ukrainian women, the strongest significant path effects were shown by: (1) Fitness Evaluation (FE)—the higher the level of internalization of the body standard, the higher the level of subjective assessment of physical fitness; (2) Overweight Preoccupation (OWP)—the higher the level of internalization, the stronger the fear of fat and weight alert, as well as the tendency to diet; and (3) Self-classified Weigh (SCW)—the higher the level of internalization of sociocultural norms regarding the athletic body ideal, the higher the subjectively perceived body weight.

The internalization of the athletic ideal had no effect on the level of appearance orientation (AO) and health orientation (HO) or on BMI.

Family pressure in Ukrainian women turned out to be the strongest direct causal effect explaining the influence of several body image variables, i.e., fitness orientation (FO), self-classified weight (SCW) and overweight preoccupation (OWP), on body image shaping. The impact of family pressure on FO, SCQ, and OWP was positive, while in the case of appearance evaluation (AE) and body area satisfaction (BAS), the effects were negative; i.e., the higher the family pressure on body image among young Ukrainians was, the lower their assessment of their appearance was and the more dissatisfied they were with certain areas of their body. In addition, the effect of family pressure on BMI was noted. Family pressure had no direct effect on the other components of body image in this group.

### Characteristics of the influence of family messages on the internalization of the standards of the athletic body (musculature) and on the general body image in the polish group

**Figure 3:**
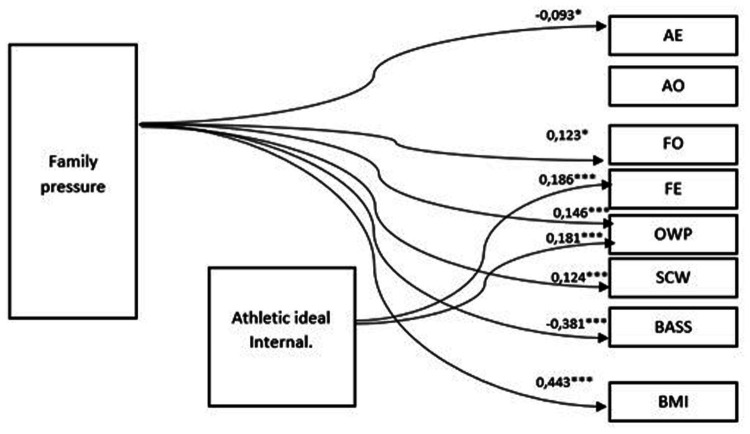
Path model (SEM) for young Polish women. *N* = 154; Chi^2^ = 3.20; df = 1; *p* > 0.05; RMSEA = 0.050; CFI = 0.994. * = *p* < 0.05; *** = *p* < 0.001 (two tailed test).

The SEM results for Polish women, and thus the model obtained as a result of the analysis (Figure 3), are comparable to those for the Ukrainian group. Again, the model fails to predict the relationship between family pressure and body image that is mediated by internalizing a tailored, more athletic standard of beauty.

For the readability of the graph, only significant paths are presented, and graphical markings of the residuals are omitted.

In regard to the influence of internalization of the athletic figure ideal, in the case of young Polish women, the results are to some extent comparable with that of Ukrainian women—significant path effects were noted for fitness evaluation (0.186) and overweight preoccupation (0.181), but there is no path connecting internalization and SCW, as was the case in the Ukrainian analysis. There was also no effect of athletic ideal internalization on BMI.

The coefficients of paths connecting family pressure with body image components in young Polish women are very similar to those in Ukrainian women. The only slightly larger difference is observed in the influence of the family on fear of being overweight, which has a lower but still statistically significant value in young Polish women (0.146 to 0.241 in the Ukrainian sample). It is also worth noting the strong positive effect of family pressure on BMI; the coefficient (0.433) is the largest of all paths included in the three tested models. The critical ratios for differences (CR) values indicated the significance of these differences.

### Characteristics of the influence of family messages on the internalization of the standards of the athletic body (musculature) and on the general body image in the Italian group

As in the case of Ukrainian and Polish women, the model describing the relationship between the variables indicates an independent, direct impact of both the internalization of the ideal of an athletic figure and of family pressure on body image components. No relationship was found between the internalization of the ideal of an athletic figure and family pressure.

**Figure 4:**
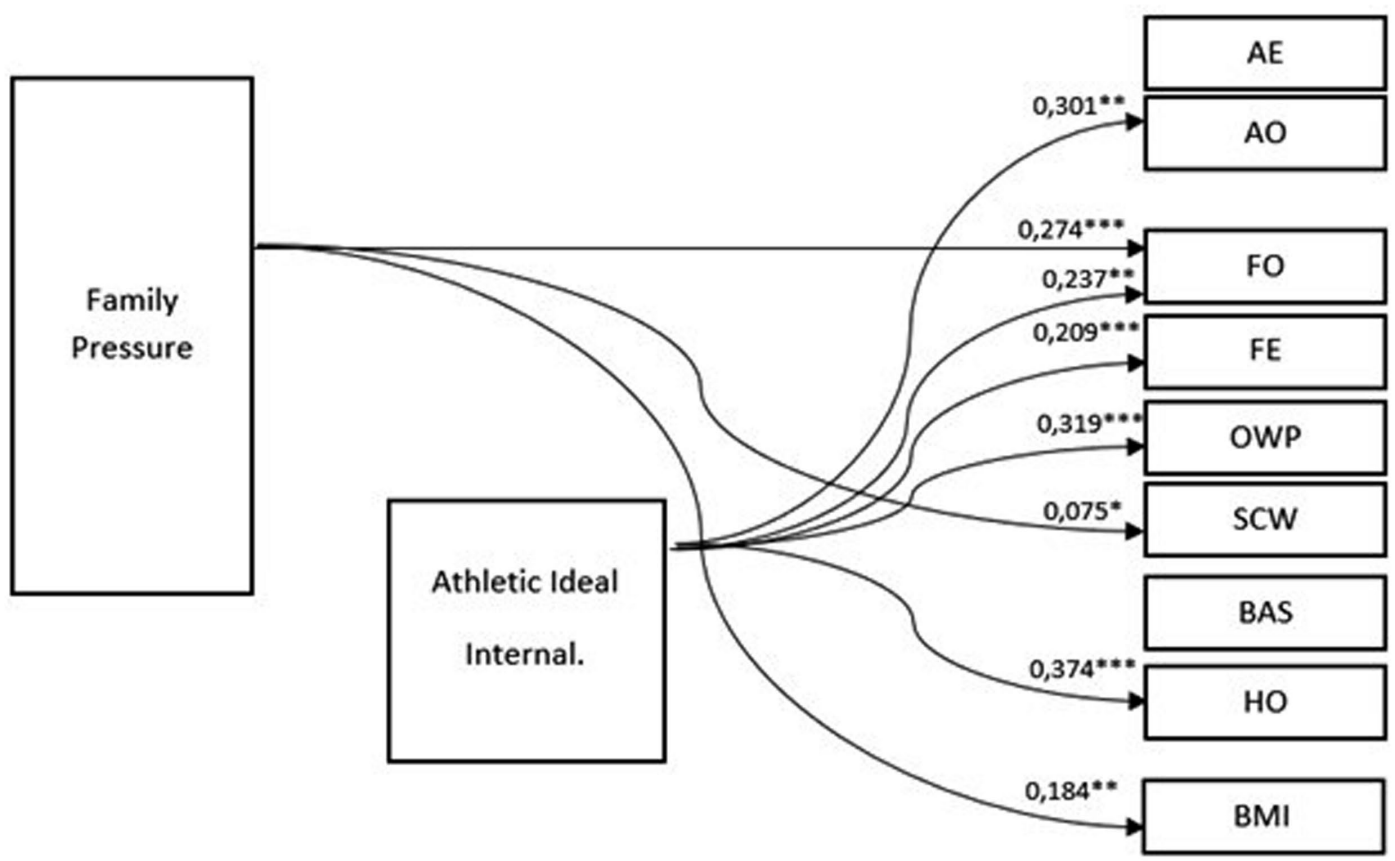
Path model (SEM) for young Italian women. *N* = 106; Chi^2^ = 0.030; df = 1; *p* > 0.05; RMSEA = 0.036; CFI = 0.994. * = *p* < 0.05; ** = *p* < 0.01; *** = *p* < 0.001 (two tailed test).

However, in the case of Italian women, there is a more comprehensive influence of the standard of beauty of the female body than in the case of Ukrainian and Polish women. As shown in Figure 4, internalization affects five components of body image: (1) health orientation (HO, 0.374), which means an increase in investment in physical body health and a healthy lifestyle as internalization increases; (2) overweight preoccupation (PLO, 0.319), indicating that greater internalization is accompanied by a greater fear of weight gain; (3) appearance orientation (AO, 0.301), which means that greater internalization of the athletic ideal can lead to greater investment in their appearance and greater importance that young Italians affix to their appearance; (4) fitness orientation (FO, 0.237), which suggests that women who recognize the standard of athletic ideal tend to actively improve or maintain their body fitness; and (5) rate their fitness higher (FE, 0.209).

For the readability of the graph, only significant paths are presented, and graphical markings of the residuals are omitted.

Unlike Ukrainian and Polish women, family pressure turned out to have a direct impact on only two components of body image: fitness orientation (FO, 0.274) and self-classified weight (SCW, 0.075). However, for the latter, the effect appears to be relatively small, while the effect on FO is greater than that reported for Ukrainian and Polish women. The path between family pressure and BMI (0.184) was also noted; its coefficient for Italian women was the lowest of all three models.

## Discussion

### Characteristics of intercultural differences between polish, Ukrainian, and Italian women in terms of body image, internalization of the ideal body musculature, and family pressure

By attempting to answer the first question about the differences between women from Ukraine, Poland, and Italy in terms of multidimensional body image, the authors obtained the results of statistical analysis indicating significant differences between the studied groups. The differences were visible in the areas of body image, the strength of family pressures and the internalization of the ideal of an athletic figure. It turned out that Polish women differ from both Ukrainian and Italian women, showing a significantly lower assessment of fitness and care for their own physical fitness, higher self-esteem of their own health than Ukrainian and Italian women, but also lower care for health than other respondents, higher level of concentration on weight and a lower level of Athletic Ideal Internalization. Among the respondents, Italian women showed the strongest level of influence of family pressure on body image, and the level of family pressure on body image was slightly lower but significantly different from the others, as was demonstrated by data related to Polish women. The lowest level of family pressure on body image was shown by Ukrainian women.

Very few studies in the literature review have results that can be compared with the research of this article. Ramme et al. (27) studied a sample of 421 Australian women aged 17–40 and measured a similar set of variables that would describe the relationship between body image, internalization of the athletic standard of beauty, and family influence; these results are not comparable to the results of analyzing groups of women from culturally different regions of Europe. The results obtained in this study can be compared only with those that were carried out in other populations and that had similar theoretical assumptions. For example, Frederick, Fessler, and Haselton (39) studied both men and women; their results showed the presence of a different and higher intensity of athletic ideal internalization among men than among women.

### Characteristics of the influence of family pressure on body image and the internalization of body muscles on the body image of women

The results of the study by the authors of this article did not confirm the significant and direct influence of family pressure on the internalization of the standards of sports body posture in any of the three study samples (Polish, Ukrainian, and Italian women). There was no evidence of a mediating role of the internalization of sports body posture standards between family pressure and the components describing the body image of the surveyed women. The assumptions of the Thompson model and using SATAQ 4 and MBRSQ to measure variables showed similar relationships between family pressure and body image and the internalization of the sports body ideal. These studies demonstrated the influence of the mass media, family, and peers on the ideal of a thin body, but the results of their research did not confirm a significant relationship between pressure from various social contexts and family or the internalization of athletic body shape in Australian women.

The studies on Australians also did not discuss the direct impact of family pressure on experiencing one’s own body, which was noted in these studies—in the population of Ukrainian, Polish, and Italian women, the existence of a relationship between the family and various components of body image was found. On the other hand, in the group of Polish and Ukrainian women, there was also a correlation between family pressure and appearance evaluation, overweight preoccupation, and body areas’ satisfaction.

The literature review mainly shows the relationship between the internalization of body musculature standards and the assessment of body image. This applies to the previously cited studies on the female population (24, 26) and the studies related to the male population. Several studies conducted in the Polish male population also indicated the main role of the musculature in the overall self-esteem and self-esteem of the male body (16). The results of the study confirming the influence of internalization of body musculature standards on the body image of women were also noted in other studies by Izydorczyk et al. (40), where a greater intensity of internalization of the athletic body ideal was noticed in men (41).

On the other hand, studies involving men from various cultural areas indicate similar relationships between family and sociocultural factors and the need to have a muscular, athletic figure. For example, Galioto et al. (15) demonstrated the relationship between negative family and peer experiences and behaviors focused on increasing body musculature. Regression analysis suggests the importance of paternal and peer modeling as well as peer encouragement as predictors of body change behavior.

Tylka (19) conducted a similar study on a group of men. In studies based on the Thompson model and that point to the relationship between pressure from the environment, assessment of own muscles and dissatisfaction with the body. In Tylka’s research, the influence of friends, family, media, and dating partners turned out to play a significant role in the development of muscle and fat dissatisfaction in the studied men. Similar results from the study were also obtained by Valls et al. (17), whose studies in the French population showed a correlation between dissatisfaction with the body and dissatisfaction with the body musculature. Depression was also considered in these studies.

Other studies also showed a significant influence of sociocultural factors on the development of idealization of a muscular and athletic figure in men. For example, the results obtained by Arbour (42) suggest that the ideal, muscular body image promoted by the media is associated with male dissatisfaction with their body. Leit et al. (43) found that, compared with the control group, male students who viewed commercials depicting muscular men noticed a greater discrepancy between their own muscle level and the muscle level they considered ideal. The studies of Tylka (19), which involved European-American, African-American, and Hispanic students, also indicated the relationship between body musculature and body satisfaction. The results of the research by Michael et al. (44) also confirm the influence of family pressure on body image in the group of girls and boys.

## Limitations and conclusion

The aim of the research described in this article was to verify the role of internalizing the ideal of an athletic figure in shaping the influence of the family on body image in young women from three European countries. As the analysis showed, the body image of Ukrainian, Polish, and Italian women depends on these two factors, but they have a direct impact on how women experience their bodies. The research also suggests significant differences in the importance of internalizing the athletic body ideal and family pressure between the three populations, which may suggest cultural differences between young women living in Eastern, Central, and Western European countries.

However, the study has some limitations. The first is related to the number of studied samples: In the study, the most numerous group was Ukrainian women, and the number of Italian women who took part was less than half the size. The fact that the questionnaire was conducted online may also impact the results of the study, since some studies show [e.g., (45, 46)] that trials in online research may differ from those traditionally included in offline research. This may suggest insufficient representativeness of the sample for the rest of the population. It seems, however, that in a situation where young people are the subject of the study, this risk is less likely.

An important factor that could influence the obtained results is the period of data collection. The study was conducted during the COVID-19 pandemic, which could have influenced many of the factors considered in the study. Some studies suggest that during a pandemic, women may experience increasing difficulties in eating regulation, preoccupation with food, and deteriorating body image (47). Therefore, it cannot be ruled out that the results obtained in the MBRSQ study may have been influenced by the context of the study.

Despite these limitations, however, the project appears to be a valuable contribution to the study of the body image of young women. The study is all the more valuable, as it indicates significant intercultural differences in the level of intensity of phenomena traditionally associated with nutritional psychopathology. This, in turn, suggests that research on risk factors and predictors of recovery disorders is insufficiently versatile and that there is a need to conduct research on these issues while considering sociocultural background.

The research may be of application significance because the results may be used in the creation of educational programs. Evidence-based prevention programs may be more positively accepted by parents. Due to the comparisons of three cultural perspectives and family factors: Italy, Poland, and Ukraine in relation to the ideal of an athletic female figure and different ways of perceiving one’s own body image, the results may be useful in building European programs for use in health prevention and working with young people on their image. Body and stimulating proper health behavior toward the body in the trend of “body positivity.”

## Data availability statement

The raw data supporting the conclusions of this article will be made available by the authors, without undue reservation.

## Ethics statement

The studies involving human participants were reviewed and approved by the Ethics Board for Research Projects at the Institute of Psychology, University of Gdansk, Poland (decision no. 33/2020). The patients/participants provided their written informed consent to participate in this study.

## Author contributions

BI, BB-K, and ML: conceptualization. SL and ML: methodology. BI and SL: formal analysis. BI, TY, NB, RO, ML, US-R, and BMR: investigation. BI, BB-K, and SL: data curation. BI, KG, BB-K, TY, NB, RO, SL, KS-W, US-R, BMR, and ML: writing—original draft preparation. BI, BB-K, TY, NB, SL, KS-W, US-R, BMR, and ML: writing—review and editing. BI, BB-K, KS-W, and ML: supervision. BI and ML: project administration and funding acquisition. All authors contributed to the article and approved the submitted version.

## Conflict of interest

The authors declare that the research was conducted in the absence of any commercial or financial relationships that could be construed as a potential conflict of interest.

## Publisher’s note

All claims expressed in this article are solely those of the authors and do not necessarily represent those of their affiliated organizations, or those of the publisher, the editors and the reviewers. Any product that may be evaluated in this article, or claim that may be made by its manufacturer, is not guaranteed or endorsed by the publisher.
